# Cracking the code: Pioneering early detection and management of breast cancer in the Brazilian public healthcare system

**DOI:** 10.1016/j.dialog.2025.100235

**Published:** 2025-08-26

**Authors:** Arn Migowski, Ruffo Freitas-Junior, Jose Bines, Angela Marie Jansen, Angélica Nogueira-Rodrigues, Maria del Pilar Estevez-Diz, Mariana Rico-Restrepo, Gayatri Sanku, André Mattar

**Affiliations:** a Division of Clinical Research and Technological Development, Research and Innovation Coordination, National Cancer Institute (INCA), Ministry of Health, Rio de Janeiro-RJ, Brazil; b Professional Master's Program in Health Technology Assessment, Education and Research Coordination, Instituto Nacional de Cardiologia (INC), Ministry of Health, Rio de Janeiro-RJ, Brazil; c CORA – Advanced Center for Diagnosis of Breast Diseases, Federal University of Goias, Goiania, GO, Brazil; d Instituto Nacional de Câncer, Rio de Janeiro, Brazil; e Instituto D'Or de Pesquisa e Ensino (IDOR), Rio de Janeiro, Brazil; f Americas Health Foundation, Washington, DC, USA; g Research Center, UFMG - Federal University of Minas Gerais, Belo Horizonte, MG, Brazil; h Brazilian Group of Studies in Breast Cancer (GBECAM), Dom Oncologia and Grupo Oncoclínicas, Belo-Horizonte, MG, Brazil; i Oncologia Clínica do Instituto do Câncer do Estado de São Paulo 'Octávio Frias de Oliveira' – ICESP, São Paulo, Brazil; j Americas Health Foundation, Bogota, Colombia; k Mastology, Hospital da Mulher SP, Oncoclinicas São Paulo, Brazil

**Keywords:** Brazil, Breast cancer, Breast cancer diagnosis, Early Detection of Cancer, Equitable access, Policy recommendations, Regional disparities, Mass screening, Health Inequities, Public Health Systems Research

## Abstract

Breast cancer (BC) remains a significant health concern in Brazil, particularly within its public healthcare system, the Unified Health System, known by its Portuguese acronym “SUS”, with early detection being one of the main challenges. A review of literature and policy documents was conducted to evaluate the performance and challenges of BC screening and early diagnosis in SUS. Brazilian experts in BC early detection attended a three-day meeting to discuss the challenges of SUS's existing early detection program and provide recommendations for surmounting them. The study identified that Brazil's current opportunistic BC screening model perpetuates issues with access to screening and regional disparities, while also generating low effectiveness and inefficiency. It also highlights several causes of delays in early diagnosis and treatment. The conclusions suggest an urgent need for an organized national BC screening program, in addition to the implementation of early diagnosis strategies, with multifaceted interventions, including urgent referral guidelines for suspected cases, training of key health professionals, patient navigation, and one-stop breast clinics. Implementing these changes could alleviate the economic strain on the healthcare system while improving patient outcomes.

## Highlights

• Brazil's opportunistic screening model limits effectiveness and efficiency.
• Low mammogram utilization worsens early detection challenges.
• Delays in diagnosis and treatment harm breast cancer patient outcomes.
• Regional disparities amplify inequities in breast cancer care.
• Reducing Brazil's cancer burden requires a multifaceted policy approach.

## 1 Introduction

Breast cancer (BC) is a significant global public health issue, and the World Health Organization's Global Breast Cancer Initiative (WHO-GBCI), established in 2021, highlights the urgent need to strengthen healthcare systems and scale services to address its increasing burden. This initiative advocates for sustainable, cost-effective, and equitable early detection and treatment services for BC, particularly in low- and middle-income countries [1].

The Brazilian College of Radiology and Diagnostic Imaging, the Brazilian Society of Mastology, and the Brazilian Federation of Gynecology and Obstetrics Association strongly support the implementation of annual mammography screening for women aged 40–74 years who have an average risk profile. Additionally, they endorse the development of tailored screening protocols for older women and those classified as high-risk [[2], [3], [4]].

In contrast, the national guidelines for breast cancer screening (BCS), introduced by the Brazilian Ministry of Health (MoH) in 2015 and updated in 2018, recommend biennial screening for women aged 50–69 and strategies to promote early diagnosis of symptomatic cases. Within the Brazilian Unified Health System (known by its Portuguese acronym SUS), which provides universal healthcare to over 200 million citizens and serves as the sole healthcare provider for 75 % of the population, BCS mammograms are offered at no cost, including to individuals outside the target population. Despite this, participation rates in the target population remain below 50 % in certain regions, significantly diverging from the MoH‘s objective of achieving a 70 % coverage rate [5,6].

Organized population-based screening, which invites all women within the target population to undergo mammograms, has been proven to reduce BC mortality [7]. However, Brazil's current approach is characterized by an opportunistic screening model, where mammograms are recommended only during medical consultations for unrelated issues, when women proactively seek screening, or as diagnostic mammograms to investigate suspicious signs and symptoms [8]. This paper aims to respond to the WHO-GBCI's “call to action” and critically examines the prevailing landscape of BC early detection and management in Brazil, proposing actionable strategies to enhance outcomes within the public healthcare system.

## 2 Methods

Americas Health Foundation (AHF) assembled a panel of six Brazilian experts in BC early detection and management who were selected for their prominence in scientific publications, their role as opinion leaders on BC control in the country, and their diverse background, including breast surgery, clinical oncology, epidemiology, public health, implementation science, and evidence based-medicine. AHF tasked each expert with writing a short paper using the literature and their experience on one of the following topics: screening and early diagnosis of breast cancer; barriers to accessing mammography; cancer policy and disease burden; treatment, and the financial burden of breast cancer in Brazil. After completing their papers, the panel convened for a three-day meeting in July 2024 to collectively analyze the data, debate, and combine their short manuscripts into a single comprehensive paper. They also discussed implementation challenges for SUS and provided recommendations to address them.

### 2.1 Role of the funding source

The organization and implementation of the workshop and manuscript preparation were carried out by AHF, a 501(c) (3) nonprofit organization dedicated to improving healthcare throughout the Latin American Region, and were supported by an unrestricted grant from MSD. MSD had no influence on the design, implementation, or content of this manuscript.

## 3 Results

### 3.1 Screening and early diagnosis of breast cancer in Brazil

Contradictory findings have emerged when comparing nationwide mammogram surveys. Data from the National Health Survey (NHS) indicate relatively high mammogram coverage across Brazil, while studies based on Health Information Systems report much lower rates. The 2019 NHS revealed that 58·3% of women aged 50–69 had undergone at least one mammogram in the prior two years, an increase from 54·3% in 2013 [9]. Additionally, the percentage of women aged 50–69 who had never had a mammogram decreased from 31·5% in 2013 to 24·2% in 2019. These NHS findings suggest that screening coverage in Brazil may be higher than in many countries included in a global cancer screening repository, only falling behind some European nations [10]. However, NHS self-reported data may be overestimated, and the discrepancies in coverage from studies based on information systems data can also be attributed to unequal coverage between SUS and the private healthcare system (both included in survey estimates) and, conversely, to incomplete data from information systems [11].

A study comparing the number of required screening mammograms to those performed by SUS in 2019 shows a 45·1 % deficit in exams needed to screen the target population, with deficits ranging from 31·4 % in the South to 70·5 % in the North [12]. The 2019 NHS indicated that regions with BCS mammograms (North, Northeast, Central-West) experienced the most significant increase since 2013, demonstrating some progress in reducing inter-regional disparities. However, inequalities persist, with coverage in the Southeast at 65·2 % compared to 43·2 % in the North [9].

**Fig. 1:**
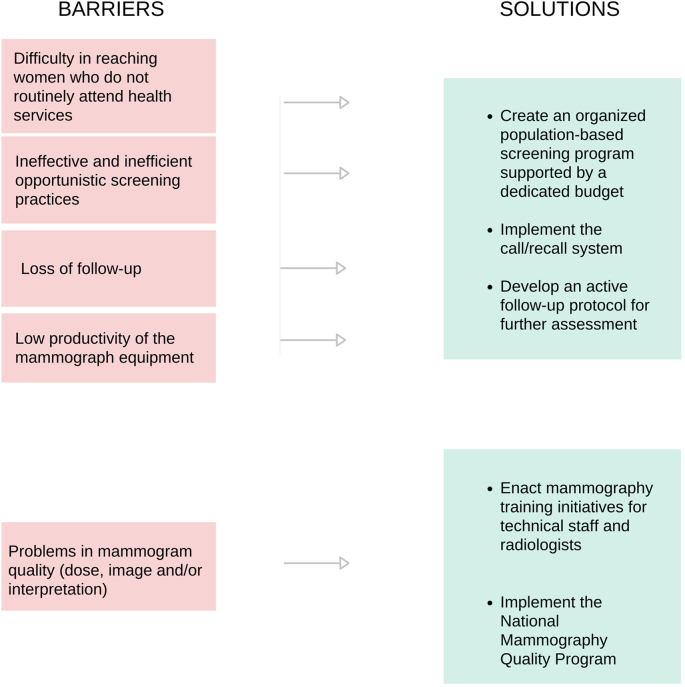
Barriers to an Effective Breast Cancer Screening and Solutions. This diagram outlines the primary barriers and corresponding solutions identified in the breast cancer screening process in Brazil.

If all SUS screening mammograms were performed on the target population, the deficit would decrease to 14·8% nationwide, with a 6·2% excess in the Southern region [13]. The latest NHS indicates that 60·0 %of women aged 40–49 had undergone a mammogram, with 43·5% through SUS and 49·7% having had one less than two years before the interview [9]. However, this is likely an overestimation. A summary of barriers and solutions to improve BCS in Brazil is presented in Fig. 1.

In Brazil, difficulties accessing healthcare have led to a significant portion of BC cases being diagnosed at advanced stages. In 2000, 41·2% of cases were identified at stages III and IV. A multicenter study highlighted that most patients were diagnosed at stages II (53·5%) and III (23·2%) [5]. Although existing cost-effectiveness models in Brazil do not consider the potential harms such as overdiagnosis and overtreatment and lead time bias effects on benefits estimation, a national study confirmed that the benefits, particularly in terms of avoided deaths, substantially outweigh these risks with biennial screening for women aged 50–69 [14]. However, the absolute benefit observed in Brazil is approximately half that estimated in the UK, using similar methods [14]. Furthermore, the impact on quality of life linked to false-positive results, overdiagnosis and overtreatment must also be considered when assessing the impact of screening [14], [16].

### 3.2 Barriers to accessing breast cancer early detection and management

#### 3.2.1 Screening

While the availability and geographic distribution of mammography equipment have improved nationwide, the primary challenge remains the underutilization of this equipment, with only 29% of capacity currently in use. This gap in utilization is predominantly due to a lack of adequately trained personnel, which is the leading factor contributing to the nationwide deficit exceeding 70% in diagnostic mammograms and biopsies, thereby severely limiting early detection of BC [15]. Addressing underutilization will require enhancements to training programs for both technical personnel and radiologists to effectively address these productivity gaps and ensure equitable access to screening services. (Fig. 1).

#### 3.2.2 Early diagnosis

Early diagnosis and prompt initiation of treatment are critical, as timely detection facilitates therapeutic interventions that significantly improve patient survival rates. The effectiveness of early diagnosis relies on the establishment of a comprehensive healthcare network, the enhancement of regulatory frameworks, and the integration of telehealth services[17]. Furthermore, population-based parameters should be used to organize the healthcare network to ensure access to diagnostic tests and treatment [18], [19]. Substantial evidence supports the connection between early diagnosis and timely treatment initiation, positively impacting patient outcomes. For instance, Denmark successfully optimized its healthcare system during the pre-screening era, significantly reducing tumor size and improving survival rates [8,16]. Although BCS remains a contentious issue due to potential harm-benefit imbalances and costs, early diagnosis strategies are essential for addressing the high incidence of advanced-stage tumors within SUS. Furthermore, advancements in adjuvant therapies have reduced the lethality associated with palpable lesions, underscoring the need for a multifaceted approach [16,18,19].

#### 3.2.3 Treatment

**Fig. 2:**
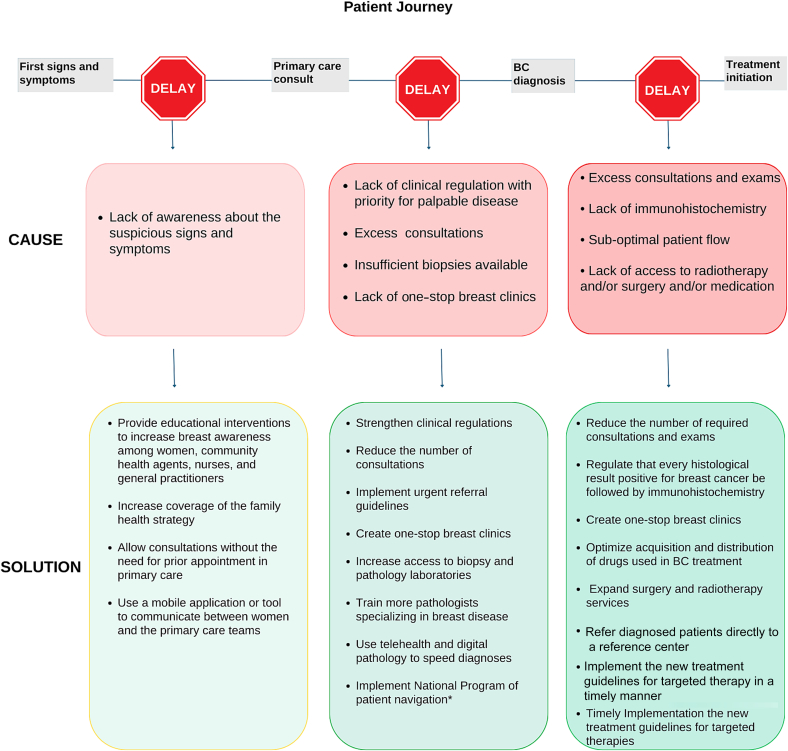
Patient Journey and Barriers to Early Diagnosis and Treatment of Breast Cancer in Brazil. This figure illustrates the patient journey and highlights the causes of delays in diagnosis and treatment along with potential solutions.

Early-stage BC treatment significantly improves outcomes. Timely interventions like surgery, radiation, and systemic therapies can effectively eradicate cancer, thereby reducing the risk of recurrence. Survival rates are significantly higher for patients diagnosed with early-stage BC. According to the American Cancer Society [20], the 5-year relative survival rate for localized BC is approximately 99%, compared to 28% for metastatic breast cancer (mBC). Furthermore, early detection often allows for less aggressive treatments, such as lumpectomy with radiation instead of mastectomy, potentially avoiding chemotherapy. This approach typically results in fewer side effects and an improved post-treatment quality of life [21]. Regarding healthcare costs, treating early-stage BC is less expensive than managing late-stage disease, as early interventions can prevent the need for more extensive and costly treatments [22]. Only 51·2% of SUS patients began treatment within 60 days after being diagnosed with BC [23] defined by law as the maximum allowable time interval [24,25]. The causes of treatment delays and proposed solutions are multifaceted, as depicted in Fig. 2.

SUS provides access to oncologic treatments, including surgery, radiotherapy, chemotherapy, hormone therapy, and select targeted therapies. In Brazil, trastuzumab is available in neoadjuvant, adjuvant, and metastatic settings for HER2 3+ patients. However, many effective therapies for higher-risk early-stage BC are unavailable through SUS. Treatments such as pembrolizumab, pertuzumab, olaparib, and TDM-1 have demonstrated survival improvements but are inaccessible in the public system, representing a gap in optimal care [[26], [27], [28]]. These treatments are only accessible to patients with private insurance coverage, despite the official incorporation of some of them. This disparity underscores ongoing challenges in equitable access to state-of-the-art BC treatments within Brazil's healthcare systems. It is also essential to consider the need for proper multidisciplinary breast cancer management to improve outcomes and successful local experiences should be encouraged and replicated [29]. Breast cancer early detection, whether by screening or improved early diagnosis, will not shift mortality curves unless correct and timely treatment is also provided.

#### 3.2.4 Cancer policy and burden of disease

Improving cancer policy and reducing the burden of BC in Brazil requires a multifaceted approach that includes regulatory and infrastructure support. The National Policy for Cancer Prevention and Control was established in 2013 [30]. A 2023 law further emphasized access to oncologic treatment and introduced the National Navigation Program for Cancer Patients, which is currently pending regulation. The National Breast Cancer Early Detection program does not officially exist within the structure of the Brazilian MoH. Although the program was created in a ministerial ordinance in 2022, it was revoked the following year. It was not approved in the tripartite inter-managerial chamber, resulting in the absence of a dedicated budget for the program [31].

Unlike some other nations, Brazil does not have a law mandating BCS programs [10] instead, there is legislation that recommends cancer screening for women and girls from puberty onwards, including BCS with mammography [32]. The National Mammography Quality Program was established in 2012 but its implementation is still incipient [5], [11]. The optimal percentage of BI-RADS® 0 mammograms is between 5 and 12 % [33] however, approximately half of all Brazilian states report mammogram rates above this desirable level [33].

The MoH Guidelines for Early Detection of Breast Cancer in Brazil currently recommend biennial mammograms for women aged 50–69 and include early diagnosis strategies for all age groups, as well as shared decision making for women aged 40–49 who seek screening [8], [68]. A federal ordinance established the Breast Cancer Diagnosis Reference Services, outlining the procedures that should be available [35]. The procedures for screening, monitoring, and diagnostic confirmation of BC within SUS are recorded in the Cancer Information System [34]. Additionally, the MoH treatment guidelines define molecular classification, staging, and treatment of BC [36]. These guidelines are not limited to treatments incorporated into SUS, leaving the decision to each service [37]. In 2024, this model of oncology guidelines was revised by the MoH, linking the recommendations to a prior assessment of their incorporation into SUS. The draft of the new treatment guidelines was presented for public consultation in February 2024 [34].

#### 3.2.5 Financial burden and socioeconomic impact of breast cancer in Brazil

The financial burden of managing BC, particularly within the Brazilian Unified Health System (SUS), stems from extensive hospitalization costs, access inequities, and significant delays in diagnosis and treatment.

BC not only imposes significant health challenges but also creates a substantial economic burden in Brazil. Approximately 70% of BC-related deaths occur among economically productive women (<69 years old), contributing to immense personal and social losses [6,38]. A recent study estimated a loss of 25·3 million years of potential productive life due to gynecological and breast cancer-related deaths between 2001 and 2030, along with a staggering US$26·8 billion loss in productivity. Nearly half of these premature deaths were attributable to BC [38].

Therefore, efforts to reduce BC mortality would result in substantial social and economic benefits. While advances in treatment have improved outcomes, the delays in diagnosis, treatment inequities, and variability in access to life-saving therapies persist, exacerbating this burden. Addressing these systemic issues offers an opportunity to improve survival rates and reduce the economic and social impacts of BC.

### 3.3 Screening and early diagnosis

The role of BCS in reducing BC mortality is well-established. An organized, population-based BCS program targeting women aged 50–69 years at regular intervals has demonstrated effectiveness in improving early-stage diagnosis and reducing mortality. Women who have never been screened would benefit the most from such programs. In Brazil, validated tools, such as a scale to predict non-adherence to screening, are available to better guide interventions [39,40].

However, the effectiveness of early diagnosis strategies is limited by geographic, socioeconomic, and systemic barriers. Regional disparities in early-stage diagnoses are stark, with rates ranging from 40·2%–53·5% in different regions [41]. In the northern, northeastern, and midwestern regions, advanced-stage diagnoses are more common, correlating with systemic inequities [41]. Mortality rates inversely correlate with metrics such as gynecologist density and the Human Development Index [41,]. This highlights the urgent need for system-wide efforts to ensure equitable healthcare access across Brazil.

Delays between diagnosis and the initiation of treatment further undermine outcomes. A study in southern Brazil revealed an average wait time of 104 days from diagnosis to first treatment, with 85·1% of surgical patients waiting over 60 days [43,44]. Conversely, efforts such as São Paulo's One-Stop Clinic, which incorporates oncology consultations at the first visit, successfully reduced wait times to less than 60 days [17]. Expanding such initiatives could significantly improve timeliness and outcomes nationwide.

### 3.4 Advancements in BC treatment

While adjuvant therapy advances have enhanced survival, they have not reduced the importance of early detection. Instead, these innovations complement early diagnosis strategies. Timely access to surgery, adjuvant therapy, and systemic treatments is critical to improving outcomes. For instance, in São Paulo, prioritized assessments for highly suspicious cases successfully demonstrate the value of reducing time-to-treatment [45].

A significant disparity exists between SUS and private healthcare systems. For example, 33·5% of SUS patients present with stage III BC, compared to just 14·7% of private healthcare patients [46,47].

As of now, post-incorporation studies evaluating the real-world impact of technologies like trastuzumab within SUS remain limited, emphasizing the need to monitor and assess new therapeutic interventions across all stages of BC [42].

### 3.5 Role of genetics and personalized medicine

Genetic testing has become increasingly important in identifying high-risk populations and guiding therapy decisions. Twenty to 30% of BC patients in Brazil carry germline mutations in high-penetrance genes such as *BRCA1* and *BRCA2* [48], [49]. Identifying these mutations has implications not only for systemic and surgical treatments but also for screening other neoplasms and offering specialized monitoring for at-risk family members.

Structured clinical genetics networks could enhance risk stratification and counseling, targeting the 1% of individuals who account for 5–10% of BC cases [7]. For example, modeling studies have demonstrated the cost-effectiveness of gene-based screening strategies for *BRCA1/2* in women with a high familial risk, suggesting favorable incremental cost-effectiveness ratios in SUS [50,51].

### 3.6 Radiotherapy and conservative treatments

Efforts to promote breast-conserving surgery and reduce mastectomy rates in Brazil remain complicated by systemic barriers and access inequities. Over the past decades, SUS has documented trends in surgical practices, including an increase in lumpectomies alongside fluctuating rates of mastectomies (with or without axillary lymphadenectomy) [52]. However, these trends are difficult to quantify due to procedural reporting constraints and the ecological design of data analysis.

Improved access to adjuvant radiotherapy may help mitigate unnecessary aggressive surgeries, further enhancing the uptake of conservative interventions. The impact on quality of life has been significant for patients treated primarily with lumpectomy or radiotherapy. For instance, patients treated at a Brazilian Oncology Reference Hospital had a mean score of 75 out of 100 [53] on the Global Health Scale of Quality of Life (WHOQOL), compared to 62 out of 100 [54] in a different radiotherapy center. These findings highlight the importance of focusing on quality of life while optimizing BC care across various institutions.

### 3.7 Sociodemographic inequities and access barriers

Sociodemographic factors such as urban residence, higher education, media access, female-headed households, health insurance, and socioeconomic status correlate with increased healthcare utilization,[40], [41]. However, access barriers persist, particularly in the underserved regions of northern, northeastern, and midwestern Brazil [41]. These disparities contribute to poorer outcomes for lower-income populations treated in SUS.

Regional differences in survival rates between the public and private healthcare systems also highlight inequities. Addressing these disparities requires strengthening public-sector access to diagnostics, early intervention, and novel treatments. For example, expanding access to preoperative oncology consultations and reducing diagnostic delays could drastically improve treatment outcomes for underserved populations.

## 4 Discussion

Herein, we presented recommendations for strategies to tackle barriers to improve BC's early detection and management in Brazil. We believe that these strategies also apply to many low- and middle-income countries, as identified in a recent systematic review [55]. Several studies conducted in Africa, Asia and Latin America demonstrate barriers to early diagnosis, such as lack of breast cancer awareness and access barriers to health services similar to those described here, as well as barriers that delay the time between diagnosis and initiation of treatment [55]. These results demonstrate that the barriers and strategies to overcome them to improve breast cancer control presented here have transferability and generalizability to other low- and middle-income countries, particularly in relation to underserved populations.

Consistent evidence from multiple studies supports the efficacy of BCS and early diagnosis in detecting early-stage BCs, thus improving overall prognosis and treatment options [56,57]. By identifying smaller, more treatable tumors, physicians can decrease the need for systemic treatment and enable less invasive surgical treatment options [50,51,[58], [59], [60], [61], [62]]. From a health economics perspective, organized screening can be cost-effective as it saves medical costs through early cancer detection and treatment, potentially reducing healthcare spending on advanced cancer treatments [63,64]. Although some early detection policies and programs exist in Brazil, implementation efforts and accountability must be improved to achieve the desired results.

**Fig. 3:**
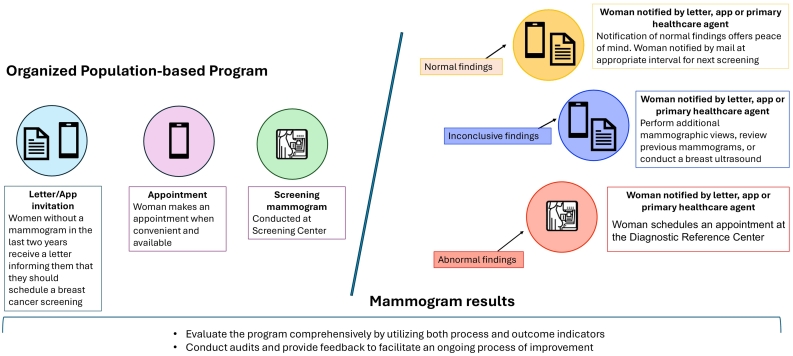
Flowchart of an Ideal Organized Population-Based Breast Cancer Screening Program in Brazil. This figure outlines the step-by-step process of an organized population-based breast cancer screening program in Brazil.

Brazil is a large country with significant regional disparities in healthcare, where access to mammography machines and treatment services varies widely across regions [65]. A recent study showed that women who participated in the last two BCS rounds before diagnosis had the largest reduction in BC deaths, while missing either of the two prior rounds significantly increased their risk [59]. The “Pink October” campaign increased mammograms performed in October and the following months, suggesting we should perform similar actions throughout the year [66]. A subgroup analysis of a recent clinical trial in India suggested the efficacy of biennial clinical breast examinations in reducing advanced-stage diagnoses and mortality rates among women over 50 in low- and middle-income countries. Given Brazil's extensive primary healthcare infrastructure, implementing a similar approach of active population targeting, qualified clinical examinations, and systematic (Fig. 3) follow-up could yield comparable improvements in BC outcomes across the nation's diverse regions [67]. Mobile screening units are acceptable options to improve access in rural areas, as long as the quality of mammograms and integration with services for diagnostic confirmation and treatment are guaranteed.[69]

Although the focus of discussions on breast cancer control in Brazil tends to be on age range recommendations and screening interval and coverage, it is essential that this debate be expanded to include essential aspects such as the quality of the entire process, including mammographic screening itself, but also diagnostic confirmation and treatment. Furthermore, it is essential to guarantee access to diagnosis and treatment, rather than focusing solely on expanding screening, and it is necessary to guarantee the comprehensiveness of the entire line of care both for the women screened and to ensure early diagnosis of women with suspicious signs and symptoms. The deficit of procedures for diagnostic confirmation is widespread in the SUS, reaching 90.8% deficit in the necessary number of core biopsies and 80.6% deficit in surgical biopsies in the central-west region and 88.5% deficit in anatomopathological exams in the north, with the impact on the diagnostic investigation of women with suspected signs and symptoms being especially worrisome [12]. This shows that it is not enough to invest only in mammographic screening, but rather to think systematically about increasing the production capacity of all these procedures, which requires medium- and long-term planning for the training and retention of specialist doctors in the health system, as well as the structuring of reference services for diagnostic confirmation.

Although several barriers described here need to be acknowledged, it is necessary to recognize that there has been a progressive improvement in stage distribution in the last two decades in the SUS, reaching a percentage of 59% of invasive breast cancers diagnosed in early stages I or II in 2019 [5], very close to the minimum standard recommended by WHO-GBCI of diagnosis at least 60% of invasive breast cancers presenting as stages I or II[70]. However, the COVID-19 pandemic had a significant impact, leading to a reduction in screening, diagnostic confirmation and surgical treatment of breast cancer in the country, causing a setback in early detection with consequences for the coming years [71].

An important limitation of this article is that the analysis performed refers to the current situation. With population aging[72] and also with increased exposure to risk factors such as obesity,[73] it is likely that the magnitude of breast cancer incidence will increase in Brazil over the next decade. This further increases the importance of prioritizing addressing the challenges and barriers presented in this article.

Another important point is the identification of research gaps and how future research could explore these aspects in more depth. In particular, research on how to best implement complex interventions that address the organization of the health system. For example, the best ways to speed up diagnostic investigation and how to avoid problems that may arise when implementing urgent referral protocols for investigating cases with suspicious signs and symptoms. Another aspect that has not been sufficiently studied is the best way for one-stop breast clinics to operate and how to implement their articulation with both primary care and oncology treatment centers. Prospective studies evaluating the implementation of screening strategies, early diagnosis and improved access to treatment should be carried out to monitor the effectiveness of these recommendations.

## 5 Conclusion

The rapidly evolving landscape of early BC diagnosis and care in Brazil underscores profound disparities and urgent challenges that must be confronted to enhance patient outcomes. Despite establishing numerous early detection policies, Brazil has yet to realize its full potential. To achieve the desired outcomes, there must be a significant improvement in the implementation process, with greater emphasis on systematic accountability and sustained efforts. Strengthening coordination between policymakers, healthcare providers, and community outreach initiatives is essential for ensuring these programs are effectively executed, reaching underserved populations and ultimately reducing the disease burden. A robust monitoring and evaluation framework will also be necessary to track progress and identify areas for improvement.

Additionally, stark regional variations in access to quality training for using existing diagnostic tools persist, particularly within SUS. These disparities are compounded by barriers between the quality of training in urban versus rural residencies and ultimately greatly influence service utilization and early-stage diagnosis rates. The findings underscore the importance of implementing comprehensive public health strategies prioritizing equitable care access. However, the current opportunistic BCS model limits the potential benefits of early detection, particularly in underserved regions. Advancements in systemic treatments offer promising avenues for personalized treatment.

Although the experts and the literature present controversies about the screening coverage figures in the country and choosing the best screening protocol, there was consensus among experts on the need to overcome several barriers both to replace the current opportunistic screening model in the country for a population-based program, as well as to overcome several barriers to advance early diagnosis strategies and better access to treatment. By prioritizing these efforts, Brazil can reduce the burden of BC.

## CRediT authorship contribution statement

**Arn Migowski:** Writing – review & editing, Writing – original draft, Visualization, Validation, Investigation, Formal analysis. **Ruffo Freitas-Junior:** Writing – review & editing, Writing – original draft, Validation, Investigation, Formal analysis. **Jose Bines:** Writing – review & editing, Validation, Investigation, Formal analysis. **Angela Marie Jansen:** Writing – review & editing, Visualization, Project administration, Methodology. **Angélica Nogueira-Rodrigues:** Writing – review & editing, Validation, Investigation, Formal analysis. **Maria del Pilar Estevez-Diz:** Writing – review & editing, Validation, Investigation, Formal analysis. **Mariana Rico-Restrepo:** Writing – review & editing, Visualization, Methodology, Conceptualization. **Gayatri Sanku:** Writing – review & editing, Project administration, Methodology. **André Mattar:** Writing – review & editing, Writing – original draft, Validation, Investigation, Formal analysis.

## Declaration of competing interest

The authors declare the following financial interests/personal relationships which may be considered as potential competing interests:

Arn Migowski reports financial support was provided by Americas Health Foundation. Ruffo de Freitas Junior reports financial support was provided by Americas Health Foundation. Maria Del Pilar Estevez-Diz reports financial support was provided by Americas Health Foundation. Mariana Rico-Restrepo reports financial support was provided by Americas Health Foundation. Angelica Nogueira Rodrigues reports financial support was provided by Americas Health Foundation. Andre Mattar reports financial support was provided by Americas Health Foundation. Jose Bines reports financial support was provided by Americas Health Foundation. Ruffo de Freitas Junior reports a relationship with AstraZeneca that includes: consulting or advisory, paid expert testimony, speaking and lecture fees, and travel reimbursement. Angelica Nogueira Rodrigues reports a relationship with AstraZeneca that includes: consulting or advisory and speaking and lecture fees. Andre Mattar reports a relationship with AstraZeneca that includes: equity or stocks. Jose Bines reports a relationship with AstraZeneca that includes: consulting or advisory. Angelica Nogueira Rodrigues reports a relationship with Brazilian Group of Gynecology Oncology that includes: board membership. Angelica Nogueira Rodrigues reports a relationship with Brazilian Society of Medical Oncology that includes: board membership. Angelica Nogueira Rodrigues reports a relationship with Latin American Cooperative Oncology Group that includes: board membership. Andre Mattar reports a relationship with Clinergy that includes: consulting or advisory. Ruffo de Freitas Junior reports a relationship with Daiichi Sankyo Inc. that includes: speaking and lecture fees and travel reimbursement. Angelica Nogueira Rodrigues reports a relationship with Daiichi Sankyo Inc. that includes: consulting or advisory and speaking and lecture fees. Andre Mattar reports a relationship with Daiichi Sankyo Inc. that includes: speaking and lecture fees. Jose Bines reports a relationship with Daiichi Sankyo Inc. that includes: consulting or advisory. Angelica Nogueira Rodrigues reports a relationship with Eisai Inc. that includes: consulting or advisory and speaking and lecture fees. Ruffo de Freitas Junior reports a relationship with FEMAMA that includes: board membership. Ruffo de Freitas Junior reports a relationship with Gilead Sciences Inc. that includes: speaking and lecture fees and travel reimbursement. Angelica Nogueira Rodrigues reports a relationship with Gilead Sciences Inc. that includes: consulting or advisory and speaking and lecture fees. Jose Bines reports a relationship with Gilead Sciences Inc. that includes: consulting or advisory. Angelica Nogueira Rodrigues reports a relationship with GSK that includes: consulting or advisory and speaking and lecture fees. Arn Migowski reports a relationship with International Agency for Research on Cancer that includes: board membership. Ruffo de Freitas Junior reports a relationship with International Agency for Research on Cancer that includes: board membership. Ruffo de Freitas Junior reports a relationship with Libbs Pharmaceutical that includes: speaking and lecture fees and travel reimbursement. Jose Bines reports a relationship with Libbs Pharmaceutical that includes: consulting or advisory. Angelica Nogueira Rodrigues reports a relationship with Eli Lilly and Company that includes: consulting or advisory and speaking and lecture fees. Andre Mattar reports a relationship with Eli Lilly and Company that includes: equity or stocks and speaking and lecture fees. Jose Bines reports a relationship with Eli Lilly and Company that includes: consulting or advisory and speaking and lecture fees. Ruffo de Freitas Junior reports a relationship with MSD that includes: consulting or advisory, paid expert testimony, speaking and lecture fees, and travel reimbursement. Mariana Rico-Restrepo reports a relationship with MSD that includes: funding grants. Angelica Nogueira Rodrigues reports a relationship with MSD that includes: consulting or advisory and speaking and lecture fees. Jose Bines reports a relationship with MSD that includes: consulting or advisory. Ruffo de Freitas Junior reports a relationship with Novartis that includes: consulting or advisory, paid expert testimony, speaking and lecture fees, and travel reimbursement. Angelica Nogueira Rodrigues reports a relationship with Novartis that includes: consulting or advisory and speaking and lecture fees. Jose Bines reports a relationship with Novartis that includes: consulting or advisory. Andre Mattar reports a relationship with Novo Nordisk that includes: equity or stocks. Angelica Nogueira Rodrigues reports a relationship with Pfizer that includes: consulting or advisory and speaking and lecture fees. Jose Bines reports a relationship with Pfizer that includes: consulting or advisory. Ruffo de Freitas Junior reports a relationship with REBRACAM that includes: board membership. Angelica Nogueira Rodrigues reports a relationship with Roche that includes: consulting or advisory and speaking and lecture fees. Andre Mattar reports a relationship with Roche that includes: speaking and lecture fees. Jose Bines reports a relationship with Roche that includes: consulting or advisory. Arn Migowski: reports a relationship with Brazilian Society of Medical Oncology that includes: board membership.

The other authors declare that they have no known competing financial interests or personal relationships that could have appeared to influence the work reported in this paper.

## Data Availability

The data used for this study will be made available on request to the corresponding author.
